# Report of Several Cases of Dipththeria

**Published:** 1861-02

**Authors:** S. T. Newman

**Affiliations:** St. Louis


### SELECTIONS.

*Report of several cases of Diphtheria.* By S. T. Newman, M. D., of St. Louis.

In the latter part of September, a family, consisting of a gentleman, his wife and two children, visited our city for the purpose of attending our State Fair. Soon after their arrival, the wife was attacked with sore throat, and I was requested to see her. The husband stated to me, that he felt serious misgivings about the character of the disease, as they were from Kentucky, where dipththeria had been, and was still prevailing in a very malignant form. Upon examining the throat, I discovered indications of a grave and ominous character; nearly the whole of the lateral aspect of the throat (involving both sides) was covered with dirty whitish patches, under which there was a tendency to ulceration of a superficial character; the entire periphery was inflamed; the tonsils were somewhat swollen and deglutition was painful; the neck was also slightly swollen on the outside. The general symptoms were anorexia, constipated bowels, coated tongue, quick pulse, with cool skin. Though these symptoms were grave enough, the uneasiness which I felt was caused more by the alarming accouuts which I had heard of the disease in Kentucky, than on account of the severity of the symptoms themselves. I at first applied to the diseased surface, by means of a large camel's hair pencil, a strong solution of nitrate of silver, and directed the bowels to be opened. I then put her upon the following ℞—Chlorate potass, ℥ iii; acid hydro-chloric, ℥ i; sulph. quinin., ℈ i; aqua dist., ℥ viii. M. Dose, a tablespoonful every two hours, or oftener. Visited the patient in the evening, symptoms unchanged. Again applied solution nitrate silver; prescription continued. On the following morning I regarded her condition as being more favorable. The pulse was, however, still frequent, numbering about 100. Treatment continued, with the addition of wine. From this period improvement continued, and the patient recovered.

While in attendance upon the mother, one of her children, a little girl about four years old, was attacked with the same disease. She was treated upon the same general principles, and recovered. Tinct. ferri muriat. was given to both during convalescence. There were no other cases in the family, or among the friends with whom they were stopping.

While attending upon these cases, a gentleman living in Stoddard Addition called on me, and desired me to see a negro girl about eleven years old, who was laboring under sore throat, and seemed quite unwell. I learned also that some friends from a distance had been stopping with him, and that some of the children had heen affected with sore throat, from which they had not fully recovered when they arrived at his house. I visited this girl, first, September 29th. Upon examining her throat, a very unfavorable condition was presented. Both sides of the throat were covered with a whitish exudation, under which there was considerable ulceration ; there was also great surrounding inflammation with swelling of the tonsils, and of course difficulty in swallowing ; articulation was hoarse and indistinct, and there was a very copious flow of saliva, as if the patient were laboring under mercurial ptyalism ; pulse frequent and small ; the bowels were constipated, tongue coated, and there was no desire for food. I could not but regard the symptoms as being of a grave character. The throat was first penciled with the nitrate of silver in solution, and then put upon the mixture above, viz : chlorate potass, quinine, and muriatic acid, with the free use of wine. The bowels were soluble by means of ext. rhei, and fluid diet, consisting of milk and broths, was ordered. The next day there was but little change in the general condition of the patient, except that there was very great external swelling of the neck.

The treatment was continued, with the addition of tinct. iodine externally applied. In the course of a day or two there was evident improvement both in the general and local symptoms; the pulse was improved; the external swelling had almost entirely disappeared; deglutition was effected with comparative ease, and articulation was more distinct; the patient slept and breathed comfortably; the ulceration, though remaining, showed indications of healing. I now felt satisfied that the patient would recover; but in a few days the ulceration began to deepen, with a tendency to sloughing, and involved not only the mucous membrane, but seemed to extend to the muscular tissue. Deglutition became very painful. The tinct. sesquichloride ferri was substituted in the mixture for the muriatic acid, and aqua chlorine was applied to the diseased surface by means of a probang, and a domestic cargle of a decoction of sanguinaria, which had been suggested, was allowed. To counteract symptoms of prostration, quinine, wine and carbonate ammonia were freely used. Swallowing soon became very difficult, and the effort produced the most violent pain in the ears, so as to cause the sufferer to place the hands to the ears and writhe in the most distressing manner. When the stimulants were regurgitated through the nose, an effort was made to rally the patient by the use of active stimulants per rectum, but without effect; the pulse became more and more feeble, and for some time before death was no longer perceptible, and yet there was no dyspnœa, and the patient appeared conscious up to the hour of dissolution, which took place October 13th.

While in attendance upon this case, the mistress began to complain of debility, with some irritation of the throat, which, upon examination, was found to be congested, but no ulcers were discernible for five or six days. As the lady is an unusually nervous, timid woman, I thought that most of her disease was imaginary, or the result of nervous debility, and prescribed sugar-coated asafœtida pills, and occasionally the mixture chlorate potass, quinine, and muriatic acid; but in five or six days, notwithstanding this treatment, several superficial ulcers were seen, covered with flocculent matter.—In some places, where these little flakes were removed, the epithelium remained unbroken; in others there was abrasion of surface. The solution of nitrate silver was applied a few times; the decoction sanguinaria was freely used as a gargle, and as the lady complained of debility wine was ordered, and the mixture containing quinine (as above) was taken more frequently. The appetite was good throughout, and nourishing diet was allowed. The patient recovered.

During the pendency of this case, a negro boy, æt. about 17, (who had attended constantly upon the girl who had died,) was observed to be dull and drowsy, and complained of sore throat, which, upon examination, was found to be considerably inflamed and swollen, but there was no ulceration or deposit of whitish flakes; his pulse was frequent and small, though he was a boy of unusual physical vigor; the tongue was coated, bowels constipated. He was put upon the mixture potass. and quinine, and was directed to use salt and water freely as a gargle; he made use of the decoction sanquinaria also for the same purpose. No nitrate silver was used. The next day, or perhaps the third day, ulcers were discovered in the throat, with the characteristic covering; there was also great tumefaction of the tonsils, with difficult deglutition, and excessive flow of saliva, and the boy was evidently greatly worse. A solution of nitrate silver was freely used, and the former treatment persevered in. During the night the gentleman himself—who was the only remaining member of the family—feeling great uneasiness in his throat, became alarmed, and while I was at the breakfast-table next morning, his brother called on me with a note expressive of great concern, and requesting me to bring Dr. Pope out with me. Upon our arrival, we found the gentleman decidedly more scared than hurt. There was indeed no cause for alarm, and he was simply ordered to use the mixture as before mentioned, and no further medication was required. The negro boy's throat was much ulcerated, and Dr. Pope discovered some effusion of lymph. The treatment was approved and continued. There was but little change in him for a day or two, except that the neck became swollen externally, and the effort to swallow—as in the case of the negro girl—caused great pain in the ears. The outside of the neck was painted with tinct. iodine, which was directed to be applied occasionally until slight burning was produced; and, upon visiting him one morning, I was surprised to find the whole anterior and lateral portions of the neck blistered, and upon one side there was a large accumulation of serum, which was discharged by clipping. No inconvenience followed the blister, but the throat rapidly improved from that time; improvement, however, was apparent before this period.

While the lady was sick she was several times visited by the wife and sister-in-law of Dr. Helms. The Doctor informs me they were both attacked with sore throat, both in a mild degree, but attended with unusual debility. In one case, for several days there was an exudation and a thin membranous deposit, which, when removed, left the mucous membrane unbroken, but again re-appeared.

It will be observed that the worst cases were confined to negroes, and that in both there was great tendency towards ulceration and sloughing. Dr. Helms has suggested to me that this is more likely to be the case in the negro; whether it be true or not, I am not prepared to say.

*Remarks.*—Upon a resume of these cases, I would remark, if there were any striking peculiarities, they consisted in the very early symptoms of debility, and the excessive pain in the ears upon attempting to swallow, which last symptom was present in two cases. In the treatment there is nothing new; it is such as is recommended in some of the European journals, except perhaps the use of iodine externally—which was suggested upon general principles—and the sanguinaria as a gargle, which was a domestic remedy. And I would here remark, Messrs. Editors, that regarding this disease as having its origin in some *materies morbi* in the blood, and the disease of the throat being only a local manifestation, I do not place much reliance in topical applications, and especially when applied with a probang, which I repudiate.—Having heard that diphtheria was prevailing with great malignancy in Lexington, Kentucky, before having seen any of the above cases, I wrote to one of the physicians of that city inquiring if there were any peculiar characteristics of the disease in that locality, and how far it was amenable to treatment, and what plan of treatment appeared best adapted, &c. Omitting to furnish any information upon general principles, he writes with some degree of self-glorification—though not in the most classic or Addisonian style—as follows, as to his treatment:

LEXINGTON, Sept. 29th, 1860.

DR. S. T. NEWMAN—*Dear Sir:* I received your letter a few days since, requesting my mode of treating *diphtheria.* In reply I would say, I give the muriate of ammonia in full doses—say, to a child eight years old and upwards, 10 grs. every two hours, (in solution,) and ten drops of the sesqui-chloride of iron in the intermediate hours, and these are not to be omitted for thirty-six hours; then rest four or five hours, and give them again in like manner. Continue this treatment for four or five days according to circumstances; but at first cleanse the stomach with a gentle purgative, if the bowels should not act, once in twenty-four hours give castor oil and ol. terebinth, ℥ i of the former to ʒ i of the latter. If the diphtheric crest forms, or has formed to a great extent in the throat, remove it with a fine sponge tied on a stick; the sponge should be wet with a solution of the pure nitrate of silver 40 grs. to ℥ i, or the sulphas cupri ℈ i to ℥ i of water; this should be used only once a day. The cure should be completed by the use of tonics; I have found beeberine the best. Diet nourishing.

I have treated three hundred and thirty-four cases after this method without the loss of one, and am now fully satisfied it is the proper mode of treating the disease.

Respectfully yours, &c.,

J. W. BRIGHT.

Since the receipt of the above letter, I have been shown a letter from a very intelligent merchant of that city, stating that the treatment of Dr. Bright has been *eminently* successful, and that he believes it will save ninety-nine out of a hundred, if not the whole.—*St. Louis Medical & Surgical Journal.*

